# Teacher–student relationship moderates the association between tuberculosis close-contact exposure and somatic symptoms among adolescents in Hangzhou, China

**DOI:** 10.3389/fpubh.2026.1921969

**Published:** 2026-08-31

**Authors:** Zijian Fang, Qingchun Li, Yinyan Huang, Qingjun Jia, Qinglin Cheng

**Affiliations:** Hangzhou Center for Disease Control and Prevention (Hangzhou Municipal Health Supervision Institute), Hangzhou, China

**Keywords:** adolescents, psychosocial stress, school health, somatic symptoms, teacher–student relationship, tuberculosis exposure

## Abstract

**Background:**

Although tuberculosis (TB) control programs have primarily focused on infection prevention, diagnosis, and treatment outcomes, increasing attention has been directed toward the psychosocial consequences of TB-related experiences among affected populations. In school settings, adolescents identified as TB close contacts may experience concerns about infection risk, repeated health monitoring, activity restrictions, and disease-related stigma, which may represent important psychosocial stressors beyond the biological risk of infection. However, evidence regarding whether TB close-contact exposure is associated with somatic symptoms among adolescents remains limited.

**Methods:**

A school-based cross-sectional study was conducted in Hangzhou, China, in April 2024. A total of 1,410 valid questionnaires were included (670 TB close contacts; 740 non-exposed students). Somatic symptoms were assessed using the Children’s Somatization Inventory-24 (CSI-24), and teacher-student relationship was evaluated using the Student-Teacher Relationship Scale (STRS). Binary logistic regression models were used to estimate associations, and interaction analyses were performed to evaluate whether the teacher-student relationship modified the association between TB close-contact exposure and somatic symptoms.

**Results:**

A total of 169 adolescents (11.99%) were classified as having elevated somatic symptoms (ESS). The prevalence of ESS was significantly higher among adolescents with TB close-contact exposure than among non-exposed adolescents (14.78% vs. 9.46%, $\chi^{2}$ = 8.925, *p* = 0.003). In univariate analysis, TB close-contact exposure was associated with increased odds of ESS (*OR* = 1.659, 95% *CI*: 1.200–2.305, *p* = 0.002). After adjustment for sociodemographic characteristics and teacher-student relationship, the association remained significant (adjusted *OR* = 1.545, 95% *CI*: 1.105–2.167, *p* = 0.011). A 10-point increase in teacher–student relationship score was associated with lower odds of ESS (*OR* = 0.861, 95% *CI*: 0.750–0.986, *p* = 0.032). Interaction analysis showed a significant interaction between TB close-contact exposure and teacher-student relationship (Per 10-point increase) (*OR* = 0.577, 95% *CI*: 0.414–0.803, *p =* 0.001), indicating that the association between TB close-contact exposure and somatic symptoms differed according to teacher-student relationship quality, with a weaker association observed among adolescents reporting more positive teacher-student relationships.

**Conclusion:**

Teacher-student relationship statistically modified the association between TB close-contact exposure and somatic symptoms. Adolescents reporting more positive teacher-student relationships showed a weaker association between TB exposure and elevated somatic symptoms. These findings suggest that the teacher-student relationship may represent an important school-based psychosocial factor associated with variability in adolescents’ responses to TB exposure. Given the cross-sectional design, the observed interaction should not be interpreted as evidence of a causal buffering effect. Longitudinal studies are needed to confirm these findings and determine whether school-based psychosocial support strategies can improve adolescent well-being during TB contact investigations.

## 1 Introduction

Although tuberculosis (TB) control strategies have substantially reduced disease burden through improved prevention, diagnosis, and treatment, school-based TB cases and contact investigations continue to occur worldwide. During these investigations, adolescents identified as TB close contacts undergo medical screening and follow-up monitoring, which may expose them to infection-related concerns, uncertainty, and psychosocial stress. Such experiences may affect adolescent health beyond infection-related outcomes through stress-related psychological and physical responses.

Somatic symptoms are common among adolescents and have been associated with impaired academic performance, reduced quality of life, increased healthcare utilization, and elevated risk of subsequent mental health problems (1). Somatic symptoms may be overlooked in school health practice because psychological distress is often expressed through physical complaints rather than overt emotional manifestations. Previous studies have demonstrated that stressful life events, health-related anxiety, and emotional difficulties contribute to somatic symptoms among young people (2). However, whether exposure to TB contact investigations is associated with increased odds of somatic symptoms among adolescents remains unclear.

According to the stress-buffering model, supportive social relationships may reduce the adverse effects of stressful experiences by enhancing perceived resources, promoting adaptive coping, and facilitating psychological adjustment (3). Therefore, identifying supportive interpersonal factors that may modify adolescents’ responses to TB-related stress is important for understanding heterogeneity in health outcomes following TB contact exposure.

The teacher-student relationship represents an important component of the school environment. Supportive teacher-student relationships have been associated with improved psychological well-being and stronger school belonging among adolescents (4). Evidence also suggests that positive teacher-student relationships may reduce vulnerability to stress-related health outcomes by providing emotional support, fostering resilience, and promoting adaptive coping strategies (5).

Previous research has largely focused on infection risk, treatment outcomes, and mental health problems among TB patients and contacts, whereas the psychosomatic consequences of TB close-contact exposure among adolescents have received limited attention. Furthermore, little is known about whether supportive school relationships may contribute to differences in adolescents’ responses to TB-related stress. To our knowledge, no previous study has examined whether teacher-student relationship modifies the association between TB close-contact exposure and somatic symptoms.

Therefore, the present study aimed to investigate the association between TB close-contact exposure and somatic symptoms among adolescents in Hangzhou, China, and to examine whether teacher-student relationship moderates this association. We hypothesized that: (1) adolescents with TB close-contact exposure would have higher odds of somatic symptoms than non-exposed adolescents; and (2) teacher-student relationship would modify the association between TB close-contact exposure and somatic symptoms.

## 2 Materials and methods

### 2.1 Study design and participants

This school-based cross-sectional study was conducted in Hangzhou, China, in April 2024. A multistage selection approach was used to identify eligible schools and participants. First, two districts/counties with reported school-based TB cases were randomly selected. Subsequently, four schools with confirmed TB index cases requiring school-based contact investigations were randomly selected from these districts/counties.

Following identification of the index TB cases, school-based contact investigations were conducted according to the national tuberculosis contact investigation criteria (6) Students who had close contact with the index TB cases, including those sharing classroom or dormitories with students diagnosed with TB (7), were identified as TB close contacts. All eligible close contacts identified during the initial investigation were invited to participate. The questionnaire survey was conducted within 2–4 weeks after identification of TB close contacts, during which exposed students had received initial health screening and routine follow-up monitoring according to local TB prevention and control procedures.

For the comparison group, non-exposed students were randomly selected from the same schools and grades as exposed students using a 1:1 matching strategy. Students were eligible for the non-exposed group if they had no documented history of TB close-contact exposure, had not participated in TB contact investigation, and had not received TB-related health monitoring during the corresponding period.

**Figure 1:**
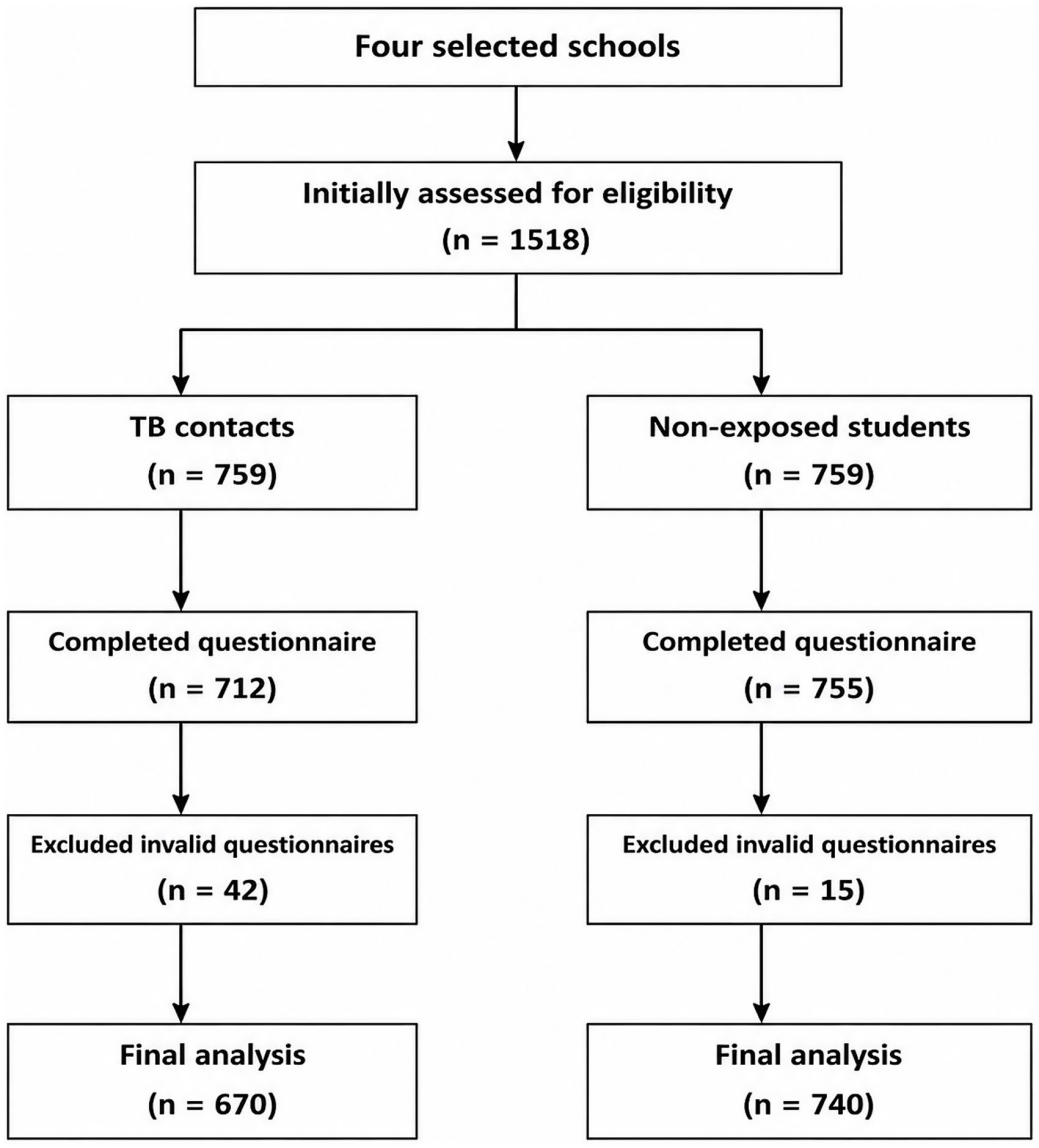
Flow diagram of participant recruitment and selection in the study.

After excluding questionnaires with substantial missing information or logically inconsistent responses, 670 exposed students and 740 non-exposed students were included in the final analysis. The participant recruitment and selection process is shown in Figure 1.

Participants were eligible if they: (1) were enrolled in the selected schools; (2) were able to complete the questionnaire independently; and (3) provided informed consent. Participants were excluded if they: (1) had severe cognitive or communication impairments; (2) had substantial missing questionnaire data; (3) provided logically inconsistent responses.

Because participants were recruited from four schools, school-level clustering was assessed using the intraclass correlation coefficient (*ICC*). The estimated *ICC* was 0.008, indicating negligible school-level variation in somatic symptoms. Given the limited number of school clusters, multilevel logistic regression was not performed, and conventional logistic regression models were used for the primary analyses.

### 2.2 Sample size calculation

Somatic symptoms were considered the primary outcome. The required sample size was estimated using a two-proportion comparison formula:


$$\;n=\frac{{\left(Z_{\alpha /2}+Z_{\beta }\right)}^{2}[P_{1}(1-P_{1})+P_{2}(1-P_{2})]}{{\left(P_{1}-P_{2}\right)}^{2}}$$

Because no previous study had specifically evaluated somatic symptoms among adolescent TB close contacts, the sample size calculation was based on an anticipated effect size. The prevalence of somatic symptoms among general adolescents was assumed to be 32.3% based on previous research (8). An odds ratio (*OR*) of approximately 1.9 was assumed based on previous studies reporting adverse psychological outcomes among TB-exposed populations (9). With a two-sided significance level of 0.05 and 80% power, the estimated minimum sample size was 298 participants. Allowing for a 10% non-response or invalid questionnaire rate, the target sample size was set at 332 participants.

### 2.3 Data collection and quality control

Data were collected using anonymous self-administered structured questionnaires in classroom settings. Before data collection, all investigators received standardized training on study procedures, questionnaire administration, and quality-control protocols. During the survey, investigators explained the study purpose and provided standardized instructions while minimizing potential influence on participants’ responses.

Completed questionnaires were checked on-site for completeness and logical consistency. Questionnaires with substantial missing data or inconsistent responses were excluded from the final analysis. All data were double-entered and cross-validated before statistical analysis.

### 2.4 Measurements

#### 2.4.1 TB close-contact exposure

TB close-contact exposure status was verified using school TB contact investigation records maintained by the local Centers for Disease Control and Prevention (CDC). All exposed participants were identified through routine school-based TB contact investigations conducted according to national TB contact investigation guidelines.

#### 2.4.2 Teacher–student relationship

Teacher-student relationship was assessed using the Student Version of the Student-Teacher Relationship Scale (STRS), a self-reported instrument designed to evaluate adolescents’ perceived relationship quality with their teachers. The STRS has demonstrated good reliability and validity among children and adolescents (10), and the Chinese version of the STRS has also shown satisfactory psychometric properties (11). In the present study, the Chinese version of the STRS (12) was used. The scale consists of 23 items assessing multiple dimensions of teacher-student relationship quality, including closeness, emotional support, communication, trust, interpersonal interaction, satisfaction, instrumental support, and conflict. Each item is rated on a 5-point Likert scale ranging from 1 (“completely disagree”) to 5 (“completely agree”). Negatively worded items were reverse scored before analysis. Total scores range from 23 to 115, with higher scores indicating more positive teacher-student relationships. The STRS demonstrated good internal consistency in the present study, Cronbach’s α = 0.885 (95% *CI*: 0.875–0.893).

#### 2.4.3 Somatic symptoms

Somatic symptoms were assessed using the Children’s Somatization Inventory-24 (CSI-24) (13), a widely used self-report instrument designed to evaluate the frequency and severity of somatic complaints among children and adolescents.

The CSI-24 consists of 24 items covering common somatic complaints, including headache, abdominal pain, dizziness, fatigue, nausea, muscle pain, back pain, and cardiopulmonary symptoms. Participants rated the extent to which they were bothered by each symptom during the previous 2 weeks on a 5-point Likert scale ranging from 0 (“not at all”) to 4 (“a whole lot”). Total scores range from 0 to 96, with higher scores indicating greater somatic symptom burden.

The CSI-24 has demonstrated satisfactory reliability and validity across pediatric and adolescent populations. In the present study, the scale showed high internal consistency (Cronbach’s α = 0.947, 95% *CI*: 0.935–0.956).

Based on previously reported cutoff criteria, participants were classified into low-risk somatic symptoms (LRSS) and elevated somatic symptoms (ESS) groups using a cutoff score of ≥19 (14, 15). In sensitivity analyses, CSI-24 total scores were additionally analyzed as a continuous outcome to evaluate the robustness of the findings.

#### 2.4.4 Covariates

Demographic variables included age, gender, school grade, ethnicity, household registration, and self-reported family economic status. These variables were considered potential confounders and were included in multivariable regression analyses based on previous epidemiological evidence and a conceptual framework regarding factors associated with adolescent somatic symptoms (16). Multicollinearity was assessed using variance inflation factors (*VIFs*), with *VIF* values <5 considered acceptable.

### 2.5 Statistical analysis

All statistical analyses were performed using *R* software version 4.4.1. Continuous variables were described as means ± standard deviations (*SD*), and categorical variables were presented as frequencies and percentages.

Differences between exposed and non-exposed groups were assessed using independent-samples *t* tests for continuous variables and chi-square tests for categorical variables. Binary logistic regression models were used to estimate odds ratios (*ORs*) and 95% confidence intervals (*CIs*) for factors associated with ESS.

Binary logistic regression analyses were conducted to examine the association between TB close-contact exposure and somatic symptoms.

Three logistic regression models were constructed sequentially. Model 1 included TB close-contact exposure as the independent variable. Model 2 was additionally adjusted for sociodemographic covariates (age, gender, grade, ethnicity, household registration, and economic status) and teacher-student relationship score. Model 3 further included the interaction term between TB close-contact exposure and teacher-student relationship score while retaining all main effects to examine effect modification.

Teacher-student relationship scores were mean-centered before generating the interaction term to reduce multicollinearity. For interpretability, the odds ratios for the main effect of teacher-student relationship and the interaction term were rescaled to represent a 10-point increase in teacher-student relationship score. This rescaling only changes the interpretation unit and does not alter the statistical model.

Simple slope analyses and predicted probability analyses were conducted to further interpret the interaction effect. Multicollinearity was assessed using variance inflation factors (*VIFs*). Model performance was evaluated using the Akaike information criterion (*AIC*), The Hosmer–Lemeshow goodness-of-fit test, and McFadden pseudo *R^2^*.

All statistical tests were two-sided, and *p* values < 0.05 were considered statistically significant.

### 2.6 Reporting guideline

The study was reported according to the Strengthening the Reporting of Observational Studies in Epidemiology (STROBE) Statement for cross-sectional studies. The completed STROBE checklist is provided in Supplementary Table 1.

### 2.7 Ethics statement

The initial TB close-contact investigation and health monitoring program was reviewed and approved by the Ethics Committee of Hangzhou CDC before participant recruitment and data collection in July 2022. The present study involved secondary analysis of anonymized data derived from this approved program and was approved by the Ethics Committee of Hangzhou CDC (Approval No. 2025-EHC-01). The study was conducted in accordance with the Declaration of Helsinki. Written informed consent was obtained from all participants and their legal guardians before data collection. All questionnaires were completed anonymously, and confidentiality was strictly maintained.

## 3 Results

### 3.1 Participant characteristics

A total of 1,410 adolescents were included in the final analysis, including 670 (47.52%) participants with TB close-contact exposure and 740 (52.48%) non-exposed participants. Overall, 169 participants (11.99%) were classified as having ESS.

The prevalence of ESS was significantly higher among adolescents with TB close-contact exposure than among non-exposed adolescents (14.78% vs. 9.46%, $\chi^{2}$ = 8.925, *p* = 0.003).

Among all participants, 882 (62.55%) were younger than 18 years, 952 (67.52%) were female, and 733 (51.99%) were junior high school students. Most participants were of Han ethnicity (98.01%), and 815 students (57.80%) reported rural household registration.

The mean STRS score was 61.39 ± 12.03. Adolescents with TB close-contact exposure had significantly lower teacher-student relationship scores than non-exposed adolescents (59.35 ± 14.74 vs. 63.24 ± 8.49, *t* = 5.993, *P* < 0.001). No significant differences were observed between the two groups in age, gender, grade, household registration, ethnicity, or family economic status.

Detailed participant characteristics are presented in Table 1.

**Table 1:** Baseline characteristics of adolescents according to TB close-contact exposure status.

Variable	Category	Exposed (*n* = 670)	Non-exposed (*n* = 740)	statistic	*P*-value
Age	<18 (years)	415 (61.94)	467 (63.11)	$\chi^{2}$ = 0.158	0.691
	≥18 (years)	255 (38.06)	273 (36.89)		
Gender	Male	214 (31.94)	244 (32.97)	$\chi^{2}$ = 0.127	0.721
	Female	456 (68.06)	496 (67.03)		
Grade	Senior high school	310 (46.27)	367 (49.59)	$\chi^{2}$ = 1.428	0.232
	Junior high school	360 (53.73)	373 (50.41)		
Household registration	Rural	396 (59.10)	419 (56.62)	$\chi^{2}$ = 0.790	0.374
	Urban	274 (40.90)	321 (43.38)		
Ethnicity	Han	658 (98.21)	724 (97.84)	$\chi^{2}$ = 0.095	0.758
	Other	12 (1.79)	16 (2.16)		
Economic status	Medium and above	546 (81.49)	617(83.38)	$\chi^{2}$ = 0.518	0.472
	Below middle	124 (18.51)	123 (16.62)		
Somatic symptoms	ESS	99 (14.78)	70 (9.46)	$\chi^{2}$ = 8.925	0.003
	LRSS	571 (85.22)	670 (90.54)		
Teacher-student relationship score	Mean±*SD*	59.35 ± 14.74	63.24 ± 8.49	*t* = 5.993	<0.001

Categorical variables are presented as *n* (%) and comparisons between groups were performed using the chi-square test. Continuous variables are presented as mean ± SD and were compared using independent-samples *t*-tests.

### 3.2 Association between TB close-contact exposure and elevated somatic symptoms

In univariate logistic regression analysis, TB close-contact exposure was significantly associated with ESS. Compared with non-exposed adolescents, those with TB close-contact exposure had higher odds of ESS (*OR* = 1.659, 95% *CI*: 1.200–2.305, *p* = 0.002).

Among demographic factors, senior high school students had lower odds of ESS compared with junior high school students (*OR* = 0.590, 95% *CI*: 0.421–0.819, *p* = 0.002). Higher teacher-student relationship scores were also associated with lower odds of ESS (*OR* = 0.981, 95% *CI*: 0.968–0.994, *p* = 0.005). No significant associations were observed for age, gender, ethnicity, household registration, or family economic status (Table 2).

**Table 2:** Univariate logistic regression analysis of factors associated with elevated somatic symptoms.

Variable	Category	*OR*	95% *CI*	*P* value
Tuberculosis close-contact exposure	Non-exposed	1.00 (Reference)	–	–
	Exposure	1.659	1.200–2.305	0.002
Age	<18 years	1.00 (Reference)	–	–
	≥18 years	0.992	0.708–1.379	0.961
Gender	Female	1.00 (Reference)	–	–
	Male	1.201	0.854–1.674	0.286
Grade	Junior high school	1.00 (Reference)	–	–
	Senior high school	0.590	0.421–0.819	0.002
Ethnicity	Other	1.00 (Reference)	–	–
	Han	1.138	0.394–4.815	0.834
Household registration	Urban	1.00 (Reference)	–	–
	Rural	0.856	0.620–1.184	0.346
Economic status	Medium and above	1.00 (Reference)	–	–
	Below middle	1.066	0.692–1.596	0.764
Teacher-student relationship	Continuous	0.981	0.968–0.994	0.005

In multivariable logistic regression analysis, TB close-contact exposure remained significantly associated with ESS after adjustment for potential confounders. Compared with non-exposed adolescents, exposed adolescents had higher odds of ESS (adjusted *OR* = 1.545, 95% *CI*: 1.105–2.167, *p* = 0.011). As shown in Table 3.

**Table 3:** Multivariate logistic regression analysis of factors associated with elevated somatic symptoms.

Variable	Category	*OR*	95% *CI*	*P* value
Tuberculosis close-contact exposure	Non-exposed	1.00 (Reference)	–	–
	Exposure	1.545	1.105–2.167	0.011
Age	<18 years	1.00 (Reference)	–	–
	≥18 years	1.037	0.723–1.477	0.843
Gender	Female	1.00 (Reference)	–	–
	Male	1.210	0.845–1.720	0.293
Grade	Junior high school	1.00 (Reference)	–	–
	Senior high school	0.613	0.437–0.856	0.004
Ethnicity	Other	1.00 (Reference)	–	–
	Han	1.132	0.385–4.835	0.843
Household registration	Urban	1.00 (Reference)	–	–
	Rural	0.826	0.594–1.150	0.256
Economic status	Medium and above	1.00 (Reference)	–	–
	Below middle	1.053	0.679–1.591	0.811
Teacher-student relationship	Per 10-point increase	0.861	0.750–0.986	0.032

Adjusted ORs were estimated using multivariable logistic regression including all variables listed in the table.

A higher teacher–student relationship score was associated with lower odds of ESS (per 10-point increase: *OR* = 0.861, 95% *CI*: 0.750–0.986, *p* = 0.032). Senior high school students also showed lower odds of ESS than junior high school students (*OR* = 0.613, 95% *CI*: 0.437–0.856, *p* = 0.004).

All covariates showed acceptable levels of multicollinearity (*VIF* range: 1.008–1.128). The correlation between age and grade was weak (*r* = 0.057) (Table 4).

**Table 4:** Variance inflation factors for covariates included in the multivariable logistic regression model.

Variable	*VIF*
TB close-contact exposure	1.053
Age	1.128
Gender	1.097
Grade	1.013
Ethnicity	1.008
Household registration	1.021
Household economic status	1.021
Teacher-student relationship	1.091

The correlation between age and grade was weak (*r* = 0.057).

### 3.3 Interaction between TB close-contact exposure and teacher–student relationship

To evaluate whether teacher-student relationship modified the association between TB close-contact exposure and ESS, an interaction term between TB exposure and mean-centered STRS scores was incorporated into the logistic regression model.

The interaction between TB close-contact exposure and teacher-student relationship was statistically significant (Per 10-point increase: *OR* = 0.577, 95% *CI*: 0.414–0.803, *p* = 0.001). Type III Wald chi-square testing confirmed the significance of the interaction term ( $\chi^{2}$ = 10.608, *p* = 0.001).

Conditional effect analysis showed that among adolescents with lower teacher-student relationship scores (−1 *SD*), TB close-contact exposure was associated with substantially higher odds of ESS (*OR* = 3.086, 95% *CI*: 1.757–5.423, *P* <0.001). The association remained significant at the mean level of teacher-student relationship (*OR* = 1.593, 95% *CI*: 1.115–2.274, *p* = 0.010), whereas no significant association was observed among adolescents with higher teacher-student relationship scores (+1 *SD*) (*OR* = 0.822, 95% *CI*: 0.497–1.358, *p* = 0.444) (Table 5).

**Table 5:** Conditional association between TB close-contact exposure and elevated somatic symptoms at different levels of teacher–student relationship.

Teacher–student relationship level	Adjusted *OR*	95% *CI*	*P*-value
Low (−1 *SD*)	3.086	1.757–5.423	<0.001
Mean	1.593	1.115–2.274	0.010
High (+1 *SD*)	0.822	0.497–1.358	0.444

**Figure 2:**
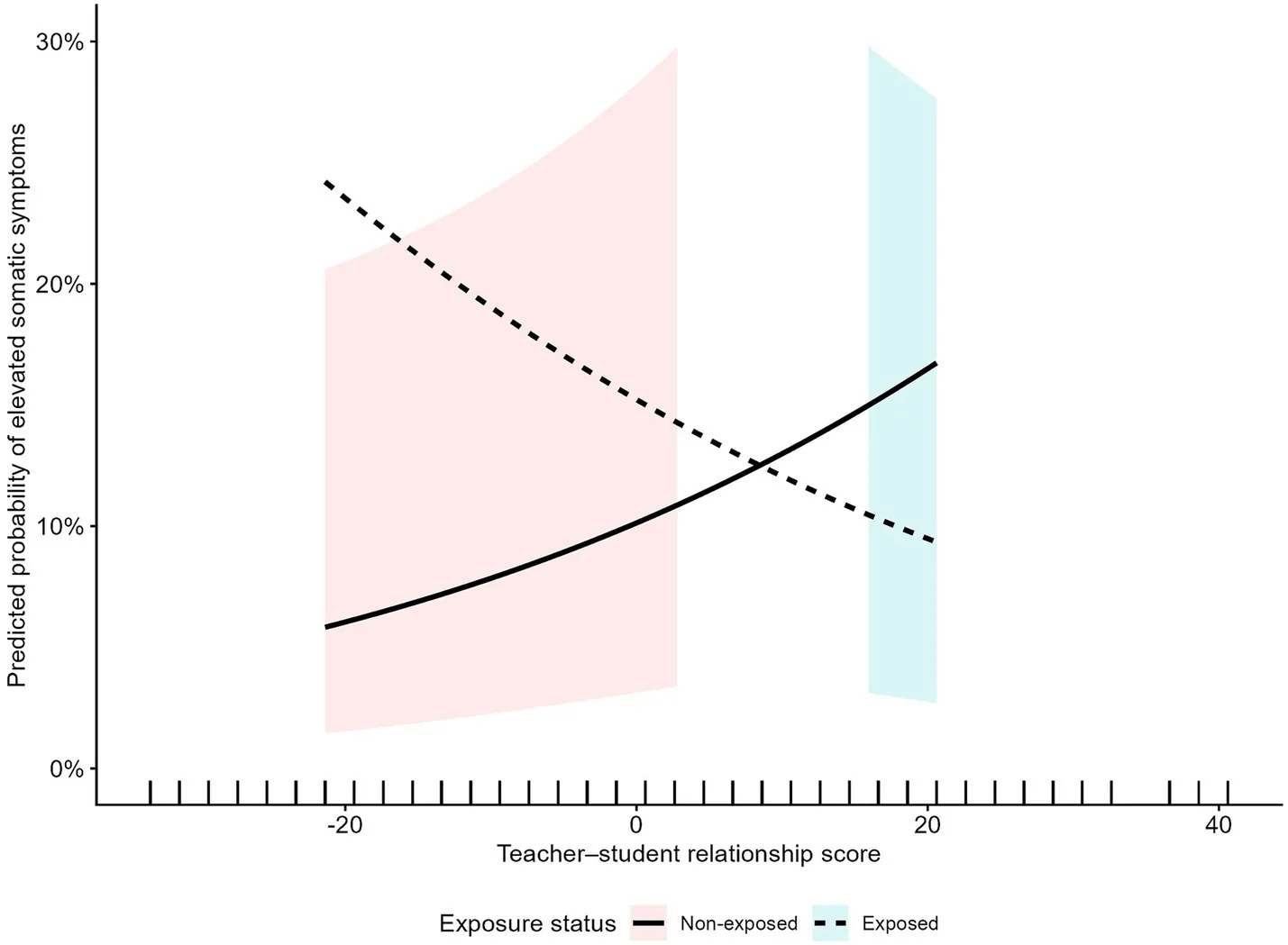
Predicted probability of elevated somatic symptoms according to mean-centered teacher–student relationship scores by exposure status.

Predicted probability analyses demonstrated that the probability of ESS decreased with increasing teacher-student relationship scores among exposed adolescents, whereas it remained relatively stable among non-exposed adolescents (Figure 2).

The interaction model showed improved model fit compared with models without the interaction term (*AIC*: 1017.6 vs. 1026.2 and 1028.5). The Hosmer–Lemeshow test indicated good model calibration ( $\chi^{2}\;$ = 4.495, *p* = 0.810), and McFadden pseudo *R^2^* was 0.035.

**Figure 3:**
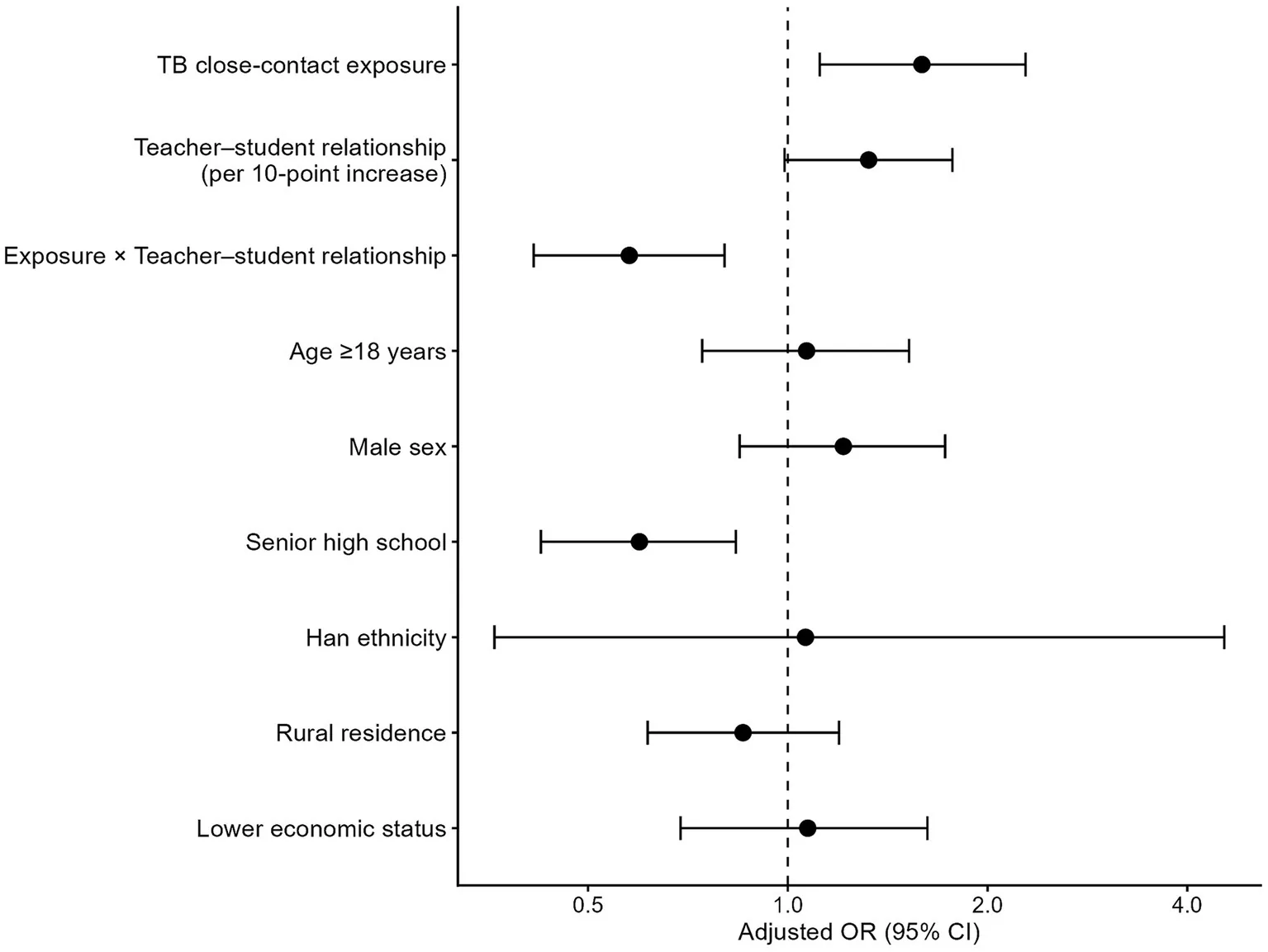
Forest plot of the moderation model for the association between tuberculosis close-contact exposure and elevated somatic symptoms.

The full interaction model is presented in Table 6, and the corresponding forest plot is shown in Figure 3.

**Table 6:** Moderation effect of teacher-student relationship on the association between TB close-contact exposure and somatic symptoms.

Variable	Category	*OR*	95% *CI*	*P*-value
Tuberculosis close-contact exposure	Non-exposed	1.00 (Reference)	–	–
	Exposure	1.593	1.117–2.282	0.010
Teacher-student relationship	Per 10-point increase	1.324	0.989–1.770	0.058
Exposure × Teacher-student relationship	Interaction term	0.577	0.414–0.803	0.001
Age	<18 years	1.00 (Reference)	–	–
	≥18 years	1.067	0.743–1.523	0.722
Gender	Female	1.00 (Reference)	–	–
	Male	1.213	0.846–1.726	0.288
Grade	Junior high school	1.00 (Reference)	–	–
	Senior high school	0.598	0.424–0.835	0.003
Ethnicity	Other	1.00 (Reference)	–	–
	Han	1.063	0.361–4.547	0.921
Household registration	Urban	1.00 (Reference)	–	–
	Rural	0.856	0.615–1.195	0.359
Economic status	Medium and above	1.00 (Reference)	–	–
	Below middle	1.072	0.689–1.623	0.750

OR, odds ratio; CI, confidence interval; Teacher–student relationship scores were mean-centered before constructing the interaction term; All variables were simultaneously included in the moderation logistic regression model.

### 3.4 Sensitivity analysis

Sensitivity analyses were conducted by treating CSI-24 scores as a continuous outcome. After adjustment for sociodemographic characteristics, TB close-contact exposure remained significantly associated with higher CSI-24 scores (*β* = 13.170, 95%*CI*: 6.275–20.066, *p <* 0.001). Furthermore, the interaction between TB close-contact exposure and teacher-student relationship remained statistically significant (*β* = −0.364, 95%*CI*: −0.583 to −0.146, *p* = 0.001). These findings were consistent with the primary logistic regression analyses based on the ESS cutoff and supported the robustness of the observed associations (Table 7).

**Table 7:** Sensitivity analysis using continuous CSI-24 scores.

Variable	*β*	95%*CI*	*P* value
TB close-contact exposure	13.170	6.275–20.066	<0.001
Teacher-student relationship	0.143	−0.045–0.332	0.135
TB exposure × teacher-student relationship	−0.364	−0.583–-0.146	0.001
Age	0.722	−0.513–1.957	0.252
Gender	0.689	−0.579–1.957	0.287
Grade	−2.797	−3.962–-1.632	<0.001
Ethnicity	0.444	−3.696–4.584	0.833
Household registration	−0.768	−1.946–0.411	0.202
Economic status	0.187	−1.343–1.717	0.810

## 4 Discussion

The present study investigated the association between tuberculosis close-contact exposure and somatic symptoms among adolescents and examined whether the teacher–student relationship modified this association. Three main findings were identified. First, adolescents with TB close-contact exposure had a higher prevalence of ESS than non-exposed adolescents (14.8% vs. 9.5%). Second, TB close-contact exposure remained significantly associated with ESS after adjustment for sociodemographic characteristics and teacher–student relationship, with exposed adolescents showing approximately 54.5% higher odds of ESS (adjusted *OR* = 1.545, 95% *CI*: 1.105–2.167). Third, teacher–student relationships significantly modified this association, such that the association between TB exposure and somatic symptoms was weaker among adolescents reporting more positive teacher–student relationships. However, given the cross-sectional design, this interaction should be interpreted as a statistical interaction rather than evidence of a causal buffering effect.

Although evidence regarding somatic symptoms among adolescent TB contacts remains limited, our findings are consistent with previous studies showing increased psychological distress, depressive symptoms, and anxiety among individuals exposed to TB contact investigations (17). Previous research has reported that household contacts of individuals with active TB may experience greater emotional distress than non-exposed populations (9). Qualitative studies have further suggested that TB contact investigations may be accompanied by infection-related concerns, uncertainty, stigma, and social isolation (18). These findings indicate that TB exposure may have psychosocial consequences beyond the risk of infection itself and may contribute to stress-related health outcomes among adolescents.

Several mechanisms may potentially contribute to the observed interaction. Adolescents identified as TB contacts often undergo repeated health examinations, monitoring, and uncertainty regarding their infection status. Such experiences may increase psychological burden and contribute to physical symptom reporting. From a biopsychosocial perspective, somatic symptoms may represent physical manifestations of emotional distress and stress responses (19). However, psychological factors such as perceived stress, stigma, and coping strategies were not directly measured in the present study; therefore, these potential mechanisms should be considered as hypotheses requiring confirmation in future longitudinal studies.

An important finding of this study was the moderating role of teacher–student relationship in the association between TB exposure and somatic symptoms. Adolescents reporting more positive teacher–student relationships showed a weaker association between TB close-contact exposure and ESS. This finding is consistent with the stress-buffering model, which proposes that supportive social relationships may be associated with variability in responses to stressful experiences. However, because stress-related psychological processes were not directly assessed, the present findings should be interpreted as supportive of, rather than confirmatory of, this theoretical framework.

Several mechanisms may potentially contribute to the observed interaction. Supportive teacher–student relationships may provide adolescents with emotional support and social resources, which could influence how they perceive and respond to stressful TB-related experiences. These processes may subsequently affect the expression of psychological distress through somatic symptoms. However, these mechanisms were not directly assessed in the present study and should be interpreted as hypotheses rather than established explanations. Future longitudinal studies incorporating measures of perceived stress, TB-related stigma, anxiety, coping strategies, and emotion regulation are needed to clarify the pathways underlying this association. Previous studies have shown that positive teacher-student relationships are associated with better student mental health outcomes, with supportive teacher-student relationships linked to reduced anxiety among students (20). These findings from previous studies provide additional contextual support for the potential role of teacher–student relationships in adolescent psychological wellbeing. The underlying mechanisms remain to be clarified in future longitudinal studies incorporating direct assessments of psychological processes.

The findings of this study have several important public health implications. First, school-based tuberculosis management should extend beyond infection surveillance and medical screening to include psychosocial assessment and mental health support. Adolescents identified as TB close contacts may require emotional support in addition to clinical follow-up. Second, routine screening for somatic symptoms and psychological distress may help identify vulnerable students at an early stage. Local CDCs, which play a central role in school-based TB contact investigations, could consider incorporating brief psychosocial assessments into routine contact management procedures. Third, strengthening teacher involvement in school health programs may represent a feasible and cost-effective strategy for improving adolescent well-being during tuberculosis contact investigations. Teacher training programs focusing on supportive communication, stigma reduction, emotional support, and psychological first aid may further enhance the potential supportive role of schools during infectious disease events. Finally, closer collaboration among schools, public health institutions, and mental health professionals may facilitate the development of comprehensive adolescent support systems.

The present study has several strengths. To our knowledge, this is among the first studies to examine the psychosomatic consequences of tuberculosis close-contact exposure among adolescents and to investigate the moderating role of teacher–student relationship. Furthermore, the study was conducted in a real-world school setting and incorporated a theoretically grounded moderation framework based on the stress-buffering hypothesis. The relatively large sample size also enhanced the statistical power and stability of the findings.

Several limitations should be considered when interpreting the findings. First, the cross-sectional design limits causal inference, and reverse causality cannot be excluded. Although TB close-contact exposure was identified before the questionnaire survey, the temporal relationship between exposure-related stress and somatic symptoms could not be fully established. Longitudinal studies with repeated assessments before and after TB contact investigations are needed to clarify the direction and dynamic changes of these associations. Second, all study variables were assessed using self-reported questionnaires, which may have introduced recall bias, reporting bias, and common method bias. In addition, although teacher–student relationship was identified as a potential moderating factor, several important psychosocial variables, including perceived stress, TB-related stigma, anxiety, depressive symptoms, coping strategies, and emotion regulation, were not directly measured. Therefore, psychological mechanisms underlying the observed association remain unclear. These potential pathways should be investigated in future studies incorporating comprehensive psychosocial assessments and longitudinal designs. Third, residual confounding from unmeasured individual, family, and school-related factors cannot be completely excluded. Although demographic and socioeconomic characteristics were adjusted for in the analyses, other factors, such as family support, previous stressful experiences, and school climate, may influence both teacher–student relationship and somatic symptoms. Fourth, participants were recruited from four schools, resulting in a hierarchical data structure. Although school-level clustering was negligible (*ICC* = 0.008), only four schools were included, limiting the feasibility of reliable multilevel modeling. Future studies involving a larger number of schools should consider multilevel approaches to better account for potential contextual effects. Finally, this study was conducted in selected schools in Hangzhou, China, which may limit the generalizability of the findings to other regions and educational settings. Multicenter studies involving diverse geographic areas and school environments are warranted to validate these findings.

## 5 Conclusion

TB close-contact exposure was associated with higher odds of elevated somatic symptoms among adolescents. Teacher-student relationship statistically moderated this association, with a weaker association observed among adolescents reporting more positive teacher-student relationships. These findings highlight the importance of considering psychosocial factors during TB contact investigations. However, longitudinal studies are required to determine causal pathways and evaluate effective school-based interventions.

## Data Availability

The datasets presented in this article are not readily available because the datasets generated and/or analyzed during the current study are not publicly available due to ethical and privacy restrictions involving adolescent participants but are available from the corresponding author on reasonable request and subject to approval by the Hangzhou Center for Disease Control and Prevention (Hangzhou Municipal Health Supervision Institute). Requests to access the datasets should be directed to chenghzcdc@sina.com.

## References

[1] Heimann P Herpertz-Dahlmann B Buning J Wagner N Stollbrink-Peschgens C Dempfle A . Somatic symptom and related disorders in children and adolescents: evaluation of a naturalistic inpatient multidisciplinary treatment. Child Adolesc Psychiatry Ment Health. (2018) 12:34. doi: 10.1186/s13034-018-0239-y, 29988308 PMC6022439

[2] Jungmann SM Wagner L Klein M Kaurin A. Functional somatic symptoms and emotion regulation in children and adolescents. Clin Psychol Eur. (2022) 4:e4299. doi: 10.32872/cpe.4299, 36397947 PMC9667419

[3] Cohen S Wills TA. Stress, social support, and the buffering hypothesis. Psychol Bull. (1985) 98:310–57. doi: 10.1037/0033-2909.98.2.310, 3901065

[4] Zhu X Zhao H Li W Wang Z. Examining associations between teacher-student relationships and adolescent well-being: the roles of school belonging, moral disengagement, and growth mindset. Int J Ment Health Promot. (2026) 28:685–705. doi: 10.32604/ijmhp.2026.078033

[5] Keane K Evans RR Orihuela CA Mrug S. Teacher-student relationships, stress, and psychosocial functioning during early adolescence. Psychol Sch. (2023) 60:5124–44. doi: 10.1002/pits.23020

[6] National Health and Family Planning Commission of the People's Republic of China Ministry of Education of the People's Republic of China School tuberculosis prevention and control work regulations (2017 edition) (2017). Available online at: http://www.moe.gov.cn/srcsite/A17/moe_943/s3285/201707/t20170727_310182.html (Accessed June 25, 2026).

[7] Peng L Mei J Hu F Xie M Liu Z Guo Y . The role of expanded close contact screening in the tuberculosis outbreak at a school in China. Front Public Health. (2025) 13:1655711. doi: 10.3389/fpubh.2025.1655711, 40977785 PMC12443705

[8] Mahirah D Lim JM Chew MSL Peddapalli N Ho CZH Marimuttu VJ . Prevalence and associated factors of somatic symptoms among adolescents in Singapore: a cross-sectional study. Ann General Psychiatry. (2025) 24:45. doi: 10.1186/s12991-025-00582-w, 40671106 PMC12265234

[9] Galea JT Chu AL Sweetland AC Jimenez J Yataco R Calderón R . Latent TB and depressive symptoms in household contacts of persons with active TB. Int J Tuberc Lung Dis. (2023) 27:682–7. doi: 10.5588/ijtld.22.0609, 37608477 PMC10443790

[10] Magro SW Hobbs KA Li PH Swenson P Roisman GI. Meta-analytic associations between the student-teacher relationship scale and students' social competence with peers. Sch Psychol Rev. (2024) 53:496–522. doi: 10.1080/2372966X.2023.2258767, 39564582 PMC11573335

[11] Chen M Zee M Koomen HMY Roorda DL. Understanding cross-cultural differences in affective teacher-student relationships: a comparison between Dutch and Chinese primary school teachers and students. J Sch Psychol. (2019) 76:89–106. doi: 10.1016/j.jsp.2019.07.011, 31759472

[12] Zou H Qu ZY Ye F. The characteristics of teacher-student relationships and its relationship with school adjustment of students. Psychol Dev Educ. (2007) 23:77–82. doi: 10.3969/j.issn.1001-4918.2007.04.014

[13] Walker LS Beck JE Garber J Lambert W. Children's somatization inventory: psychometric properties of the revised form (CSI-24). J Pediatr Psychol. (2009) 34:430–40. doi: 10.1093/jpepsy/jsn093, 18782857 PMC2722132

[14] Stone AL Walker LS Heathcote LC Hernandez JM Basch MC Wilson AC . Somatic symptoms in pediatric patients with chronic pain: proposed clinical reference points for the children's somatic symptoms inventory (formerly the children's somatization inventory). J Pain. (2019) 20:932–40. doi: 10.1016/j.jpain.2019.02.005, 30771592 PMC6689439

[15] Yuanyuan Z Guo L Junfeng H Bohui G Xumei W . Revision and reliability and validity evaluation of the Chinese version of the children's somatization inventory. J Int Psychiatry. (2015) 42:9–12.

[16] Díez-Suárez A Hernández-González C. Somatization in childhood and adolescence: a guide to facilitate its understanding. Anal Pediatr. (2025) 102:503711. doi: 10.1016/j.anpede.2025.503711, 39952854

[17] Pan SW Yen YF Feng JY Su VYF Kou YR Su WJ. The risk of depressive disorder among contacts of tuberculosis patients in a TB-endemic area: a population-based cohort study. Medicine (Baltimore). (2015) 94:e1870. doi: 10.1097/MD.0000000000001870, 26512600 PMC4985414

[18] Mlambo LM Milovanovic M Hanrahan CF Motsomi K Morolo MT Mohlamonyane MM . The impact of ethical implications intertwined with tuberculosis household contact investigation: a qualitative study. PLoS One. (2026) 21:e0306848. doi: 10.1371/journal.pone.0306848, 41911256 PMC13035131

[19] Kozlowska K Scher S Helgeland H. Functional Somatic Symptoms in Children and Adolescents: A Stress-System Approach to Assessment and Treatment. Cham: Palgrave Macmillan (2020).

[20] Salter D Neelakandan A Wuthrich VM. Anxiety and teacher-student relationships in secondary school: a systematic literature review. Child Psychiatry Hum Dev. (2025) 56:1870–88. doi: 10.1007/s10578-024-01665-7, 38446364 PMC12628381

